# A Hybrid Finite Volume and Extended Finite Element Method for Hydraulic Fracturing with Cohesive Crack Propagation in Quasi-Brittle Materials

**DOI:** 10.3390/ma11101921

**Published:** 2018-10-09

**Authors:** Chong Liu, Zhenzhong Shen, Lei Gan, Tian Jin, Hongwei Zhang, Detan Liu

**Affiliations:** 1 State Key Laboratory of Hydrology-Water Resources and Hydraulic Engineering, Hohai University, Nanjing 210098, China; zhzhshen@hhu.edu.cn (Z.S.); a@hhu.edu.cn (H.Z.); yyshen@hhu.edu.cn (D.L.); 2 The College of Water Conservancy and Hydropower Engineering, Hohai University, Nanjing 210098, China; 161302030005@hhu.edu.cn

**Keywords:** arbitrary crack propagation, hydraulic fracturing, finite volume method (FVM), extended finite element method (XFEM), quasi-brittle materials

## Abstract

High-pressure hydraulic fractures are often reported in real engineering applications, which occur due to the existence of discontinuities such as cracks, faults, or shear bands. In this paper, a hybrid finite volume and extended finite element method (FVM-XFEM) is developed for simulating hydro-fracture propagation in quasi-brittle materials, in which the coupling between fluids and deformation is considered. Flow within the fracture is modelled using lubrication theory for a one-dimensional laminar flow that obeys the cubic law. The solid deformation is governed by the linear momentum balance equation under quasi-static conditions. The cohesive crack model is used to analyze the non-linear fracture process zone ahead of the crack tip. The discretization of the pressure field is implemented by employing the FVM, while the discretization of the displacement field is accomplished through the use of the XFEM. The final governing equations of a fully coupled hydro-mechanical problem is solved using the Picard iteration method. Finally, the validity of the proposed method is demonstrated through three examples. Moreover, the fluid pressure distribution along the fracture, the fracture mouth width, and the pattern of the fracture are investigated. It is shown that the numerical results correlated well with the theoretical solutions and experimental results.

## 1. Introduction

Fluid-driven fracture is a common yet complex multi-field physics problem. When high-pressure fluids such as water enter into an existing crack, the fracture propagation criterion is met ahead of the fracture tip, which leads to fluid-driven fracture initiation and propagation [1]. As a consequence, hydraulic fracturing (HF) has to be considered in engineering studies, such as hydraulic engineering, petroleum exploration and nuclear waste storage in deep layers [2]. For instance, ultra-high concrete dams of 200–300 m or even more have been built all over the world, those dams have a potential risk of hydraulic fracturing due to the structure damage cracks on dam surfaces. Over time, cracks are completely filled with the high-pressure water. Another important application of HF is related to enhance the well production from underground reservoirs [3,4]. Hence, it is necessary to complete research and understand the influence of hydraulic fracturing.

Since the 1950s, numerous theoretical models regarding hydraulic fractures in rock masses have been proposed in the literature, beginning with the pioneering works of Perkins and Kern [5] and Nordgren [6], who proposed and developed a theoretical model referred to as the PKN model on the basis of Sneddon’s plane strain crack propagation. Geertsma and de Klerk [7] and Khristianovic and Zheltov [8] first proposed the well-known plane strain Khristianovic–Geertsma–de Klerk (KGD) model to solve hydraulic fracturing problems, especially in petroleum engineering. These early models provided analytical solutions for hydraulic fractures, and an overview of these models was given by Adachi et al. [4]. Additionally, various studies have analyzed the mechanics of concrete hydraulic fracturing since the 1980s. Bazant [9,10,11] concluded that the distribution of fluid pressure inside the fracture is the most important factor concerning HF in concrete. Brühwiler and Saouma [12] studied the water pressure distribution along the fracture by performing hydraulic fracturing tests on concrete specimens. Gan et al. [13] conducted HF experiments on concrete specimens that had a single crack, and they obtained the water distribution within a mode I crack.

Considering the limitations of analytical models and experiments, various numerical models in the literature have been developed for the simulation of fractures in two- and three-dimensional situations, such as the finite element method (FEM), meshless methods (MMs) boundary element method (BEM), phase method [14,15] and the smoothing gradient damage model [16]. The smoothing gradient damage model can precisely capture the softening in quasi-brittle materials, but this method needs to apply to 3D and coupled problems. Boone and Ingraffea [17] proposed a numerical approach based on the finite element/difference method to analyze poroelastic materials, which allow fluid leakage within the medium surrounding the fracture. Simony and Secchi and Schrefler et al. [18,19] simulated cohesive crack growth using the FEM with mesh adaptation. Sarris and Papanastasiou [20] utilized a FEM approach that included cohesive element modelling of hydraulic fractures in a permeable material. Segura and Carol [21] used the FEM with zero-thickness interface elements to simulate the hydro-fracture flow in pre-existing discontinuities. A similar model with an additional degree of freedom for the pressure within the fracture was developed by Carrier and Grant [22]. Unfortunately, these classical FEM models that were adopted to simulate the discontinuities require advanced remeshing algorithms and they also need to maintain the mesh structure as the fracture propagates in space and time. Moreover, these models also restrict the crack path to the element edges (boundaries) or along a pre-defined path called the cohesive layer. However, MMs have been introduced to overcome these drawbacks. According to an overview given by Belytschko et al. [23], MMs can be distinguished in four main categories: (1) kernel-based methods [24]; (2) moving least square methods (MLS); (3) particle-based methods [25]; and (4) the partition of unity method (PU) [26]. The treatment of this essential boundary is not straightforward in the kernel methods and MLS because they do not satisfy the Kronecker delta property, and defining the shape function is a hard, complex task. Particle-based methods require a significant amount of computational time due to the large amount of particles required to determine the fracture criteria during the update process. To decrease the computational time, Aliabadi et al. [27] and Ganis et al. [28] illustrated that a semi-analysis method called BEM could be used to model HF in poroelastic mediums, which can therefore be used to obtain the crack opening width and the fluid leakage rate. However, BEM cracks only propagate along the pre-defined edges and they require remeshing algorithms, similar to FEM. The extended finite element method (XFEM), which is based on PU, has many of the same advantages of MMs while alleviating their negative sides.

The XFEM was first implemented by Belytschko et al. [29] and Moës et al. [30] in solid mechanics, and it has more advantages than the classical FEM fracture models, especially for a fracture that grows in an arbitrary direction without remeshing. The XFEM has been applied to many engineering areas including cohesive cracks [31,32,33], dynamic crack propagation [34,35], and thermo-mechanical fracture [36]. However, the coupled fluid–solid analysis based on the XFEM was not initially introduced until the last 10 years. The hydro-mechanic coupling behavior was investigated in [37] by adding an extra flow degree of freedom (dof) in a similar manner to the displacement dof. Réthoré et al. [38] proposed a cohesive crack model to study HFs. The XFEM model with cohesive propagation for quasi-brittle materials has been studied by researchers, see [39,40,41]. These studies considered the fluid pressure inside the fracture as an additional dof, which led to increases in both computational costs and iteration difficultly. To avoid oscillations of physical fields at the interface and obtain a meaningful solution, Wang et al. and Zhou et al. [42,43] simulated HFs by combining the XFEM and the finite volume method (FVM) in concrete dams and in tight gas reservoirs, respectively, but they used linear elastic fracture mechanics (LEFM) in the crack fracture zone. In this work, we describe a numerical method to model the hydraulic fracturing behavior in quasi-brittle materials; for this purpose, a hybrid FVM and XFEM approach with a cohesive zone model (CZM) was proposed. XFEM was developed to capture the discontinuities in the medium, which can avoid the computational task of remeshing and guarantee the crack propagation arbitrarily, compared with the classic FEM framework. Additionally, the reason for using FVM rather than FEM is that FVM can handle flow within fractures easily due to has a simpler numerical formulation. Moreover. It has the ability to conserve mass in each control cell, with the merit that variations in fracture volume can be easily depicted by variations in fracture volume in each fracture element. Fracture propagation is governed by a cohesive law for the non-linear behavior in the CZM.

This paper is organized as follows. Section 1 presents background knowledge about the HF process, including analytical solutions and numerical models. Section 2 gives the set of equilibrium equations used to describe the HF problem in quasi-brittle materials, and the proposed approach is introduced. Section 3 presents three examples of applications of the FVM-XFEM method, which highlights its ability to simulate 2D HF problems including the well-known KGD test case, HF testing of concrete specimens, and the implementation of HF in a concrete gravity dam. Finally, in Section 4, we draw some main conclusions.

## 2. Methodology

### 2.1. Conceptual Model

HF is a complex multiphase field problem, involving several components: (1) the mechanical deformation of the solid phase; (2) fluid flow within the fracture; (3) the fracture propagation; (4) fluid leakage in the medium surrounding the fracture. Here, the deformation model uses the equilibrium equation of the structure, and the crack discontinuities in the domain is handled based on the XFEM technique. The fracture flow model is modelled using FVM based on lubrication theory for an incompressible fluid [44]. It is worth mentioning that the fracture is filled with fluid without a lag region. The fracture propagation criterion is based on the cohesive crack model for the non-linear fracture processes in the crack tip [20]. However, the fourth process is not taken into account due to consideration only of low permeability materials. The deformation model and the fracture flow model are coupled through the fracture width and the fluid pressure acting on the fracture surface.

### 2.2. Deformation Model

**Figure 1:**
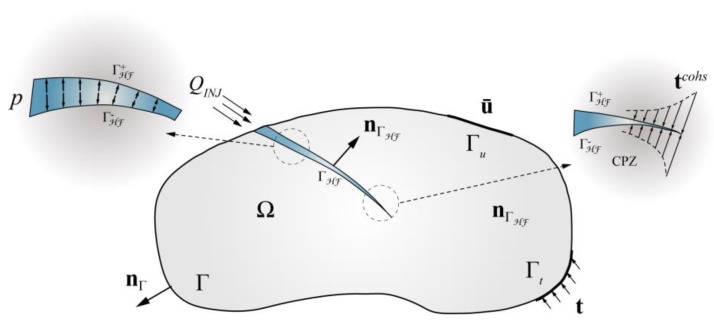
The definition and boundary conditions of a hydraulic fracturing body within a geomechanical discontinuity.

As previously described, a 2D analysis domain Ω containing an arbitrary fluid-driven fracture Г_HF_ is depicted in Figure 1. Considering that the deformation model satisfies the condition of equilibrium, the linear momentum balance for this problem can be expressed as:

(1) ∇⋅σ+ρb=0

where ***σ*** is the stress tensor, *ρ* is the body density, and **b** is the body force per unit volume. The boundary conditions of this balance equation can be expressed as:

(2) $$\{\begin{array}{l} u=\bar{u}\;\;\;\;\;\;\;\;\;\mathrm{on}\;\Gamma_{u}\\ \sigma n_{\Gamma }=t\;\;\;\;\;\;\mathrm{on}\;\Gamma_{t}\\ \sigma n_{\Gamma_{\mathrm{HF}}}=t^{\mathrm{coh}}-pn_{\Gamma_{\mathrm{HF}}}\;\mathrm{on}\;\Gamma_{\mathrm{HF}} \end{array}$$

where **n**_Г_ is the unit outward normal vector to the external boundary, $n_{\Gamma_{\mathrm{HF}}}^{-}$ and $n_{\Gamma_{\mathrm{HF}}}^{+}$ are the unit outward normal vectors on either side of the discontinuity (the + and − superscripts represent two sides of the discontinuity), *p*^+^ and *p*^−^ are the fluid pressures within the fracture surface on either side of the discontinuity, and **t***^coh^* is the cohesive traction acting at the fracture process zone. Considering that the difference between the corresponding values at the two fracture surfaces is small, it is, therefore, assumed that the fluid pressures and cohesive tractions are equivalent at both faces of the crack (i.e., *p^+^ = p^−^ = p*, *n^+^ = n*^−^ *= n*).

The linear elastic constitutive law is used to characterize the mechanical behavior of the quasi-brittle materials, which is written as:

(3) dσ=D:dε

where **D** is the fourth-order linear elastic stiffness tensor of the solid materials and ***ε*** denotes the related strain tensor; the latter can be linked to displacement by:

(4) $$\varepsilon =\frac{1}{2}(\nabla u+{\left(\nabla u\right)}^{T})$$

where **u** represents the displacement vector of the domain.

The weak form satisfying the aforementioned boundary conditions is obtained by the principle of virtual work; this can be expressed as:

(5) $$\int_{\Omega }\delta \varepsilon :\sigma d\Omega +\int_{\Gamma_{\mathrm{HF}}}⟦\delta u⟧\cdot (t^{\mathrm{coh}}-pn_{\Gamma_{\mathrm{HF}}})d\Gamma =\int_{\Omega }\delta u\cdot \rho bd\Omega +\int_{\Gamma }\delta u\cdot td\Gamma$$

where δu is the virtual displacement, ⟦δu⟧ denotes the fracture opening width across the discontinuities defined as $⟦\delta u⟧=\delta u^{+}-\delta u^{-}$.

### 2.3. Fracture Flow Model

Considering that the fracture length is much greater than the fracture width, the fracture flow model can be considered as a one-dimensional flow for simplicity [44]. Accordingly, the FVM introduced by Versteeg and Malalasekera [45] was used to calculate the fracture pressures. Compared to current models, which obtain the flow pressure distribution based on the enriched component of the fluid pressures in the XFEM, this FVM can easily and directly handle fracture flow problems.

The mass conservation of the one-dimensional incompressible fluid in the fracture can be expressed as follows:

(6) $$\frac{\partial w}{\partial t}+\frac{\partial q}{\partial x}+g=0$$

(7) $$w=⟦u_{N}⟧=⟦u⟧\cdot n_{\Gamma_{\mathrm{HF}}}$$

where *w* is the fracture opening width, *q* is the fluid flow rate, and *g* is the fluid leakage in the medium surrounding the fracture. In this instance, the fluid leakage is assumed to be zero.

Based on the equation of fluid motion inside a fracture [46], the relationship between the crack width and the fluid pressure is described as:

(8) $$q=-\frac{w^{3}}{12\mu }\frac{\partial p}{\partial x}$$

where *x* is the direction of fracture length, *p* is the pressure along the fracture, and *μ* is the fluid viscosity. By substituting the equation of motion (Equation (8)) into the mass conservation equation (Equation (6)), the obtained lubrication equation can be expressed as:

(9) $$\frac{\partial w}{\partial t}=\frac{1}{12\mu }(w^{3}\frac{\partial p}{\partial s})$$

The boundary condition for the fluid injection point can be specified as the flux or pressure boundary condition:

(10) $$\{\begin{array}{l} w(l,t)=0\\ \begin{array}{l} q(l,t)=0\\ q(0,t)=q_{0}\;\mathrm{or}\;p(0,t)=p_{0} \end{array} \end{array}$$

As previously mentioned, the fluid lag region was neglected, and the crack width was zero at the crack tip. Again, it is assumed that there is no flow at the crack tip.

### 2.4. Fracture Propagation Model

The constitutive mechanical behavior in the cohesive zone is characterized by a traction-separation law as:

(11) $$t^{\mathrm{coh}}=t^{\mathrm{coh}}\left(⟦u⟧\right)$$

The linearization of Equation (11) results in an incremental form, which can be expressed as:

(12) $$dt^{\mathrm{coh}}=D^{\mathrm{coh}}d⟦u⟧$$

**Figure 2:**
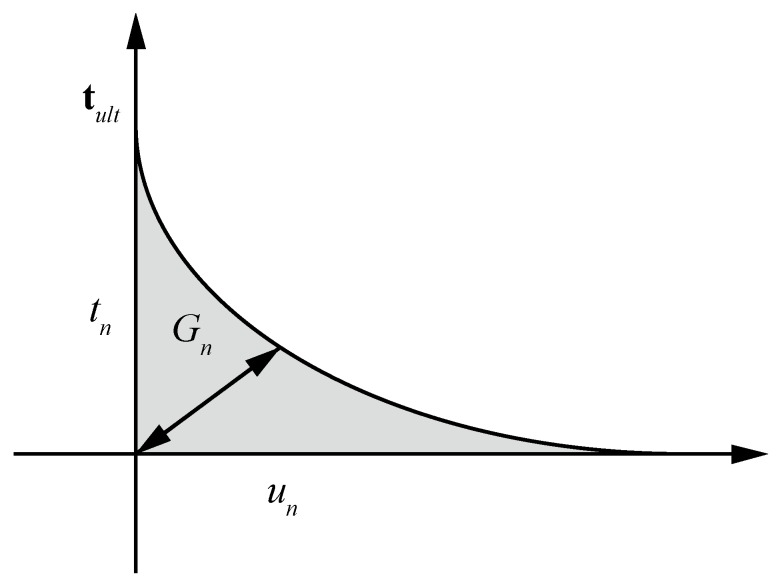
Cohesive softening law.

The traction-separation law denotes the relationship between the interface tractions and the relative displacements. Note that it is assumed that the HF process leads to displacement that exclusively occurs in the normal direction. Hence, we use an exponential softening law that is related solely to the normal opening *w*, as shown in Figure 2. Note that the cohesive traction acting at the fracture process zone can be expressed as:

(13) $$t^{\mathrm{coh}}=\tau_{\mathrm{ult}}\exp(-\frac{w\tau_{\mathrm{ult}}}{G_{c}})$$

where *τ_ult_* is the ultimate strength of the material and *G*_c_ is the unit fracture energy.

The propagation criterion of HF is satisfied when the maximum traction at the crack-tip is greater than the cohesive strength, which can be expressed as:

(14) $$t_{\max}=\begin{array}{c} \max\\ \theta \in (-\pi ,\pi ) \end{array}t_{\mathrm{eq}}=t_{\mathrm{eq}}(\theta_{\max})\geq t_{c}$$

As the stress state in the vicinity of the crack tip varies intensely, the average stress at the tip is used to obtain the smooth principal stresses based on a Gaussian weighting function [47]; the formula of the Gaussian averaging method is:

(15) $$\sigma_{tip}=\frac{\int_{\Omega }\omega \sigma d\Omega }{\int_{\Omega }\omega d\Omega }$$

where *ω* is the Gaussian weighting function; this function can be defined as:

(16) $$\omega =\frac{1}{{\left(2\pi \right)}^{3/2}a^{3}}\exp(-\frac{r^{2}}{2a^{2}})$$

where *r* is the distance to the crack tip and *a* denotes the size of the influence region of the stress, which determines how quickly the weight function decays away from the crack tip. The parameter *a* is commonly related to either Hillerborg’s characteristic length [48] or the element size. Following Dias-da-Costa et al. [49], we use approximately 1% of Hillerborg’s characteristic length, which can be expressed as:

(17) $$l_{c}=\frac{E}{1-v^{2}}\frac{G_{f}}{f_{t}^{2}}$$

Note that this characteristic length also identifies the size of the fracture process zone. Moës and Belyteschko [30] suggest using a minimum of two cohesive elements to accurately compute the distribution of tractions in this zone.

### 2.5. Discretization and Solution Algorithms

#### 2.5.1. Discretization of Equilibrium Equation

**Figure 3:**
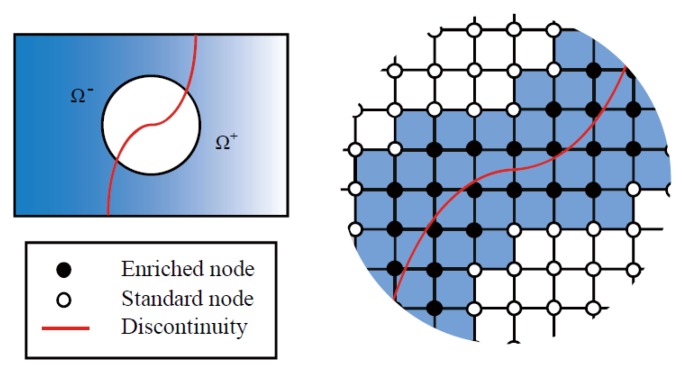
The enriched nodal points of a two-dimensional finite element including a discontinuity.

To capture the discontinuities in a HF, the displacement jumps across the fracture must be considered in the displacement field. The XFEM has been widely utilized for modelling discontinuities without the need of remeshing in the FEM framework. In the XFEM, discontinuities in the displacement field are not only enriched by an approximate function, such as the Heaviside function, for displacement jumps across the fracture, but also by an asymptotic function for the singularity displacement of the fracture tip. As shown in Figure 3, an arbitrary discontinuity divides the body into two domains, Ω^+^ and Ω^−^. At any one time, the displacement field consists of two parts: a standard displacement and an additional displacement. It is assumed that the discontinuity must cross an element without accounting for the fracture tip singularity because the crack-tip singularity will disappear once the cohesive crack model is adopted anywhere in the mesh [50]. The XFEM-based displacement approximation can thus be written as:

(18) $$u^{h}(x,t)=\sum_{I\in N}N_{I}(x)u_{I}(t)+\sum_{I\in N^{\mathrm{HF}}}N_{I}^{\mathrm{HF}}(x)H_{\Gamma_{\mathrm{HF}}}(x){\tilde{u}}_{I}^{\mathrm{HF}}(t)$$

where N is the complete nodal set, $N^{\mathrm{HF}}$ is the set of the enriched nodal points associated with the fracture, *N_I_* is the regular shape function of node *I* corresponding to the regular dofs of the displacement field **u***_I_*, $N_{I}^{\mathrm{HF}}$ is the enriched shape function of node *I* associated with the enriched dofs of the displacement field ${\tilde{u}}_{I}^{\mathrm{HF}}$, and $H_{\Gamma_{\mathrm{HF}}}$ is the Heaviside step function typically used to characterize the strong discontinuities. Note that the Heaviside step function can be expressed as:

(19) $$H_{\Gamma_{d}}(x)=\{\begin{array}{c} 1,\;x\in \Omega^{+}\\ 0,\;x\in \Omega^{-} \end{array}$$

To retain the Kronecker delta property for the blended elements that are not cut by discontinuities, a shifted enrichment Heaviside function is used that does not alter the basis of the approximation [51,52]. The displacement field in Equation (18) with the shifted enrichment can be written as follows:

(20) $$u^{h}(x,t)=\sum_{I\in N}N_{I}(x)u_{I}(t)+\sum_{I\in N^{\mathrm{HF}}}N_{I}^{\mathrm{HF}}(x)(H_{\Gamma_{\mathrm{HF}}}(x)-H_{\Gamma_{\mathrm{HF}}}(x_{i})){\tilde{u}}_{I}^{\mathrm{HF}}(t)$$

The compact form of this displacement field can be expressed as:

(21) $$u^{h}(x,t)=N(x)U_{I}(t)+{\tilde{N}}^{\mathrm{HF}}(x){\tilde{U}}^{\mathrm{HF}}(t)$$

where **N**(**x**) is the matrix of the regular shape functions corresponding to the regular dofs of the displacement field **U** and ${\tilde{N}}^{\mathrm{HF}}(x)$ denotes the matrix of the enriched shape functions associated with the enriched dofs of the displacement field ${\tilde{U}}^{\mathrm{HF}}$.

The discrete form of this equation can be generated based on the XFEM approximation (Equation (11)) using the following formation:

(22) Ku=F

where **K** and **F** denote the global stiffness matrix and the global force vector, respectively, and **u** represents the displacement vector.

(23) $$u^{e}=u_{i}+{\tilde{u}}_{i}$$

The global stiffness matrix can be divided into four categories as follows:

(24) $$K=\left[\begin{array}{cc} K_{\mathrm{uu}} & K_{u\tilde{u}}\\ K_{\tilde{u}u} & K_{\tilde{u}\tilde{u}}+T_{\tilde{u}\tilde{u}} \end{array}\right]$$

where:

(25) $$K_{\mathrm{uu}}=\int_{\Omega }B_{u}^{T}DB_{u}d\Omega$$

(26) $$K_{u\tilde{u}}=\int_{\Omega }B_{u}^{T}DB_{\tilde{u}}^{\mathrm{HF}}d\Omega$$

(27) $$K_{\tilde{u}u}=\int_{\Omega }{\left(B_{\tilde{u}}^{\mathrm{HF}}\right)}^{T}DB_{u}d\Omega$$

(28) $$K_{\tilde{u}\tilde{u}}=\int_{\Omega }{\left(B_{\tilde{u}}^{\mathrm{HF}}\right)}^{T}DB_{\tilde{u}}^{\mathrm{HF}}d\Omega$$

(29) $$T_{\tilde{u}\tilde{u}}=\int_{\Omega }{\left(N_{\tilde{u}}^{\mathrm{HF}}\right)}^{T}D^{\mathrm{coh}}N_{\tilde{u}}^{\mathrm{HF}}d\Omega$$

Note that B in the equations above is the derivative of the shape function, and the matrix components of $B_{u}$ and $B_{\tilde{u}}^{\mathrm{HF}}$ under 2D plane-strain conditions can be calculated by:

(30) $$B(x)=LN(x)\;\;\;{\tilde{B}}^{\mathrm{HF}}(x)=L{\tilde{N}}^{\mathrm{HF}}(x)$$

where **L** is the regular differential operator as defined in the classic FEM.

The global force vector is defined as:

(31) $$\begin{array}{l} F_{\mathrm{ext}}=\int_{\Gamma_{t}}N_{u}^{T}td\Gamma +\int_{\Omega }N_{u}^{T}bd\Omega ,\;\;\;\;\;\;F^{\mathrm{coh}}=\int_{\Gamma_{\mathrm{HF}}}N_{u}^{T}t^{\mathrm{coh}}d\Gamma ,\\ F_{\mathrm{ext}}^{\mathrm{HF}}=\int_{\Gamma_{t}}{\left(N_{\tilde{u}}^{\mathrm{HF}}\right)}^{T}td\Gamma +\int_{\Omega }{\left(N_{\tilde{u}}^{\mathrm{HF}}\right)}^{T}bd\Omega ,\;F^{\mathrm{HF}}=\int_{\Gamma_{\mathrm{HF}}}N_{u}^{T}pn_{\Gamma_{\mathrm{HF}}}d\Gamma \end{array}$$

**Figure 4:**
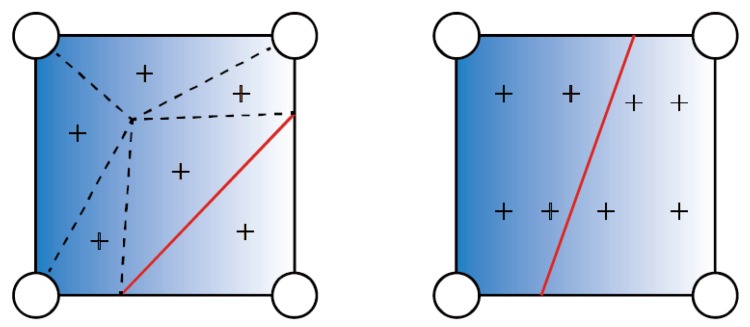
Numerical integration of an enriched element.

For these elements crossed by a discontinuity, a special treatment for the ordinary Gauss quadrature rules is required to accurately integrate the enrichment function. Figure 3 shows a finite element domain that is bisected by the discontinuity. A four-node structure mesh is used in this paper. The black nodes on both sides of the discontinuity are enriched by the additional dofs. The discontinuity within an element is assumed to be a straight line, and the fracture tips are located at an element edge. In addition, the fracture can grow in arbitrary directions, and it is also allowed to propagate through multiple elements at each time step. The standard Gauss quadrature integration is used to carry out the numerical integration in the XFEM. However, the conventional Gauss integration points are insufficient due to the arbitrary positions of the discontinuity, which may result in a loss of accuracy. To overcome this difficulty, an integrating approach introduced by Wells and Sluys is used for elements crossed by the discontinuity. In this method, additional integration points on each side of the discontinuities are generated to integrate the discretized local balance equations, as shown in Figure 4.

#### 2.5.2. Discretization of the Fracture Flow

**Figure 5:**
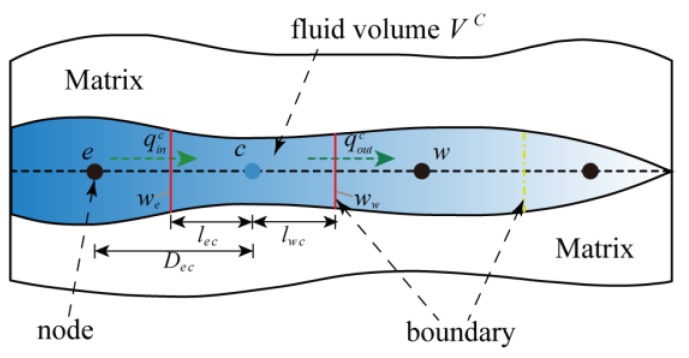
Fluid cells within a fracture using the finite volume method.

To model fluid flow within the fracture, the finite volume method was used to approximate the one-dimensional pressure field. As shown in Figure 5, the fracture segment is divided into discrete control volumes (fluid volumes). To avoid ambiguity, the discrete control volume is called a “cell”. The weak form of the lubrication equation (Equation (9)) can be written as:

(32) $$\begin{array}{l} \int_{\Delta V}\frac{\partial }{\partial x}\left(k\frac{\partial p}{\partial x}\right)dV+\int_{\Delta V}\frac{\partial w}{\partial t}dV\\ =\oint_{A}n\left(k\frac{\partial p}{\partial x}\right)dA+\int_{\Delta V}\frac{\partial w}{\partial t}dV\\ ={\left(kA\frac{\partial p}{\partial x}\right)}_{e}-{\left(kA\frac{\partial p}{\partial x}\right)}_{w}+\frac{\Delta \bar{w}}{\Delta t}\Delta V=0 \end{array}$$

where *A* represents the facial area of the control volume, *V* is the fluid volume, and *k* is the permeability of the cell, which is expressed below in Equation (33). It is worth noting that fluid pressure is assumed to be constant in each control volume.

(33) $$k=-\frac{w^{3}}{12\mu }$$

According to the Equation (32), the discretized equation of a typical finite volume element that contains a node *c* could be obtained based on the finite volume method; this results in the following representation for every cell *c*:

(34) $$T_{e}(p_{e}-p_{c})-T_{w}(p_{c}-p_{w})+\frac{\Delta \bar{w}}{\Delta t}\Delta V=0$$

where:

(35) $$T_{i}=\frac{A_{i}k_{i}}{D_{ic}}\;\;(i=e,w)$$

By applying linear interpolation, *k_i_* and *A_i_* can be expressed as:

(36) $$\begin{array}{l} k_{i}=\beta k_{w}+(1-\beta )k_{c}\\ A_{i}=\beta A_{w}+(1-\beta )A_{c}\\ \beta =\frac{l_{ic}}{D_{ic}} \end{array}$$

where *D_ic_* is the distance between the control cell *c* and its neighbouring cell *i*, and *l_ic_* is the distance between the centroid of the interface and the centroid of the control cell *c*. It is worth noting that the face area *A_i_* is replaced by the fracture aperture *w_i_* for a 2D cell.

Equation (34) can be rewritten as:

(37) Mp=W

where **M** is the stiffness matrix of the pressure field defined as follows:

(38) $$M=\left[\begin{array}{cccc} M_{1} & 0 & \cdots & 0\\ 0 & M_{2} & \cdots & 0\\ \vdots & \vdots & \ddots & \vdots \\ 0 & 0 & \cdots & M_{n} \end{array}\right]$$

In Equation (38), the cell *c* can be expressed as:

(39) $$M_{c}=[\begin{array}{ccc} m_{wc} & m_{cc} & m_{ce} \end{array}]$$

where:

(40) $$m_{wc}=T_{w},\;m_{cc}=-T_{w}-T_{e},\;m_{ce}=T_{e}$$

It is worth mentioning that the matrix *M*_1_ and *M_n_* only consist of two terms as the boundary conditions at the fluid injection point and the crack tip are known. Hence, the integration of (34) over the first control volume cell can be written as:

(41) $$m_{11}p_{1}+m_{12}p_{2}=-m_{01}p_{0}-\frac{\Delta {\bar{w}}_{1}}{\Delta t}\Delta V_{1}$$

Similarly, as far as the last control volume cell is concerned, the equation can be described as:

(42) $$m_{nn}p_{n}+m_{nn-1}p_{nn-1}=-m_{nn+1}p_{t}-\frac{\Delta {\bar{w}}_{n}}{\Delta t}\Delta V_{n}$$

where *p*_0_ is the injection pressure at the crack mouth and *p_t_* is the pressure of the crack tip, which for this case is zero, i.e., *p_t_* = 0.

### 2.6. Fluid–Solid Coupling Procedure and the Picard Iteration Approach

As discussed above, the HF is defined as a fully coupled fluid–solid interaction problem. In this process, the fluid pressure on the fracture surface causes the opening and propagation of a fracture, and the pressure is linked to the fracture flow. In turn, the fracture flow is affected by the opening and propagation of the fracture, which leads to a change of pressure within the fracture. Therefore, to properly solve this problem, a method combining FVM and XFEM can be proposed. The discretized equations of both the displacement field and the fluid field can be written as:

(43) $$\{\begin{array}{c} Ku=F\\ Mp=W \end{array}$$

The precise solution of the coupled equations above can be solved by applying the Picard iteration technique. The most common Picard iteration approach [53] can be written as follows:

(44) $$\{\begin{array}{l} p_{k+1/2}=M{\left(w_{k}\right)}^{-1}W(w_{k})\\ p_{k+1}=\alpha p_{k+1/2}+(1-\alpha )p_{k}\\ u_{k+1}=K^{-1}F(p_{k+1})\\ u_{k+1}\to w_{k+1} \end{array}$$

**Figure 6:**
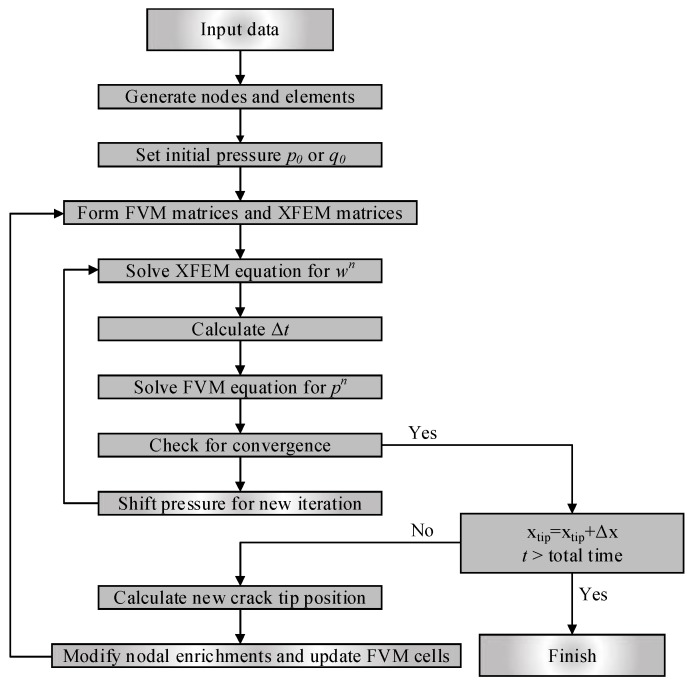
Flow chart of the finite volume and extended finite element method (FVM-XFEM) method.

In this work, the average difference of the pressure between two neighbouring iteration steps is defined to check convergence. The difference is iteratively checked until the specified convergence tolerance is reached; this can be expressed as:

(45) $$\frac{\sum_{i=1}^{N}\left|p_{i}^{\left(n\right)}-p_{i}^{\left(n-1\right)}\right|}{\sum_{i=1}^{N}\left|p_{i}^{\left(n\right)}\right|}\leq \varepsilon$$

where $p_{i}^{\left(n\right)}$ is the pressure within cell *i* during iteration *n*. The flowchart shown in Figure 6 is provided to illustrate the fluid–solid coupled process.

## 3. Results and Discussion

In this section, three examples were considered. In the first two examples, the proposed FVM-XFEM model was validated. In the third example, the FVM-XFEM model was applied to simulate the HF of a concrete gravity dam. In the first example, the well-known KGD benchmark was presented to illustrate the capabilities of the FVM-XFEM model, and the numerical simulation was compared with the analytical solution for HF propagation. In the second example, HF tests on concrete specimens with centralized cracks were carried out, and the relative terms of HF were compared between the numerical results and test results. In the third example, the FVM-XFEM model was used to study the propagation behavior of a pre-existing fracture in a concrete dam under different water levels.

### 3.1. Khristianovic–Geertsma–de Klerk (KGD) Model

In this example, the numerical results of a KGD fracture growth were presented using the same analytical information as Carrier et al. [22], and they were compared to the analytical solution introduced by Adachi et al. [4].

**Figure 7:**
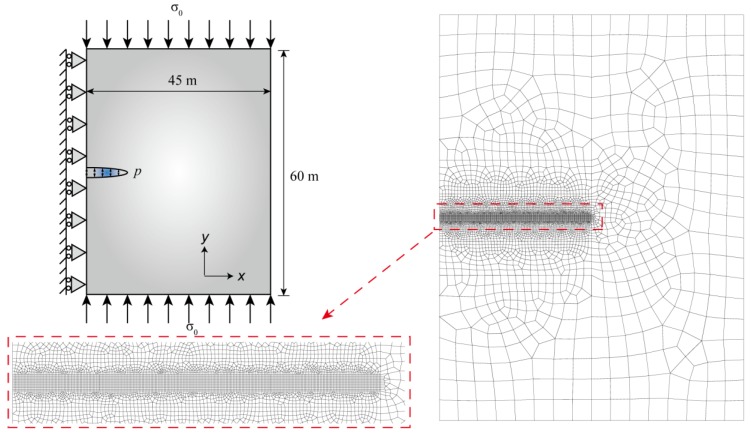
A hydraulic-driven fracture propagation in an imperious domain. A schematic representation of the Khristianovic–Geertsma–de Klerk (KGD) problem, the geometry, the boundary conditions, and the finite element mesh.

A hydraulic fracturing process in an infinite impermeable rock material was simulated based on the FVM-XFEM model. The model dimensions and boundary conditions are depicted in Figure 7. A pre-existing discontinuity with an initial length of 1 m was placed at the center of a symmetrical model, which was 60 m tall and 45 m wide. An incompressible Newtonian fluid was injected into the opening of the discontinuity with a constant rate *Q*_0_, and the fluid drove the fracture propagation. In consideration of the in situ stress state field, a confining far-field external force *σ*_0_ = 3.7 MPa was vertically imposed at the top and bottom of the model, and the left side was constrained in the *x*-direction. As previously mentioned, the linear elastic constitutive law was considered, and the cohesive crack model was used in the fracture process. The material parameters used in this analysis are given in Table 1.

The discretized information of the domain consisted of both coarse and fine meshes, and the size of each fine element was 0.1 m × 0.1 m. The refined elements in the direction of the propagating fracture were used to guarantee solution convergence and to acquire the mesh independent results because the fine meshes were necessary to resolve the pressure gradient within the cohesive zone. The area away from the potential fracture path was discretized with coarse meshes to avoid cumbersome computational calculations. For the temporal discretization, a time step ∆*t* = 0.01 was used.

An approximate analytical solution for this case without fluid leak-off and zero toughness was derived by Geertsma and de Klerk [7]. This case was called the KGD model and the analytical solutions can be expressed in the following form:

(46) $$\{\begin{array}{c} CMOD=1.87{\left(\frac{\mu (1-v)Q^{3}}{G}\right)}^{1/6}t^{1/3}\\ CMP=1.135{\left(\frac{G^{3}Q\mu }{{\left(1-v\right)}^{3}L^{2}}\right)}^{\frac{1}{4}}+S\\ L/2=0.68{\left(\frac{GQ^{3}}{\mu (1-v)}\right)}^{\frac{1}{6}}t^{\frac{2}{3}} \end{array}$$

where *CMOD* is the crack mouth opening displacement, *Q* is the injection rate of the fracturing fluid, *t* is the injection time, *μ* is the fluid viscosity, *L*/2 is the half-length of the crack, *CMP* is the crack mouth pressure, and *S* is the in situ stress.

**Figure 8:**
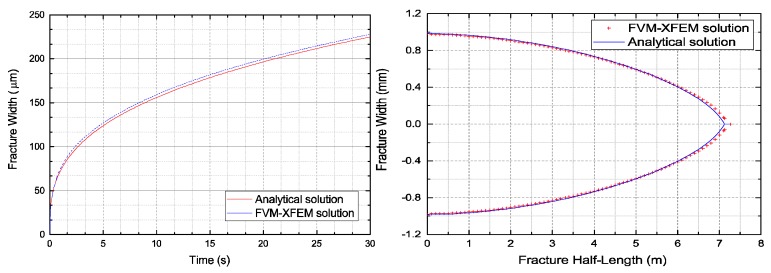
Comparison of the numerical and analytical solutions regarding: (**left**) the fracture width at the crack mouth and (**right**) the fracture width profile.

**Figure 9:**
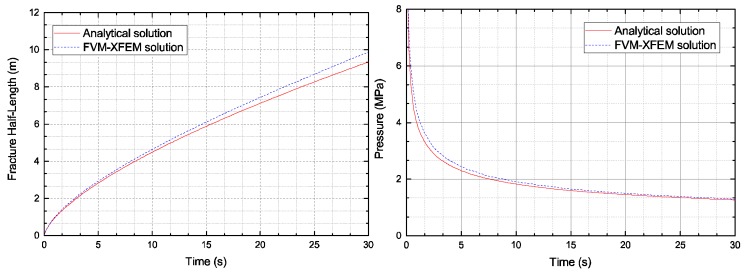
Comparison of the numerical and analytical solutions regarding: (**left**) the fracture half-length and (**right**) the fracture mouth pressure.

Figure 8 and Figure 9 compare the crack opening displacement, the crack length, and the crack mouth pressure. A good correlation was observed between the simulations presented by the FVM-XFEM model and the analytical solutions of the KGD model. From these results, it is clear that the fracture width profile was symmetrical and that the crack half-length was approximately 7 m at 20 s, which correlated well with the analytical solution. Furthermore, results confirmed the numerical results represented by Carrier and Granet using the FEM with zero-thickness cohesive elements.

**Figure 10:**
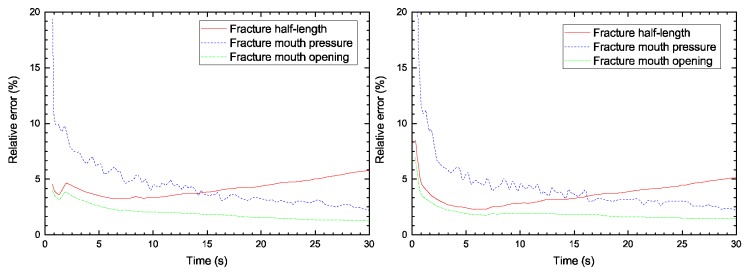
Relative error for the fracture width, the fracture half-length, and the fracture mouth pressure using: (**left**) the FVM-XFEM method and (**right**) the Carrier et al. [22] model.

In Figure 10, the relative error between the numerical solutions and the KGD analytical results is displayed. The numerical solutions contained the present model and the FEM model used by Carrier and Granet. As the results indicate, a high relative error was found at the beginning of the injection, which could be linked to the fracture process zone. As previously discussed, the characteristic length in the cohesive zone was constant. At the very first time step, the fracture length was either shorter or equal to the characteristic length. However, during the fracture propagation, a relative error reduction was observed. We attributed this small error to the differences between the analytical solution and the numerical formation. Indeed, the propagation criterion used for the two numerical models was based on the cohesive softening law, whereas linear elastic fracture mechanics was used for the analytical model. However, the relative error was almost identical between the two numerical models. The results indicate that the proposed model seemed to be equally as efficient at simulating the HF problem in the rock material as the FEM model with interface elements.

### 3.2. Hydraulic Fracturing Test

**Figure 11:**
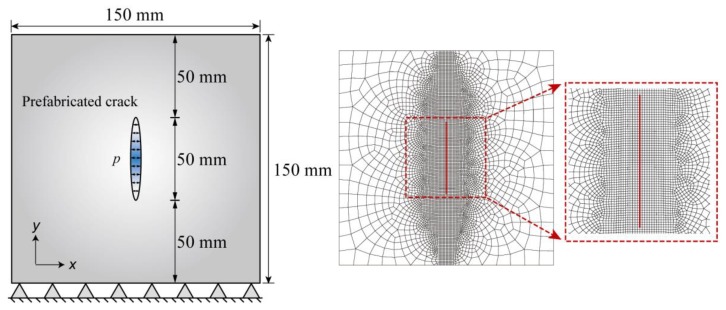
A schematic illustration of the hydraulic fracturing (HF) test: (**left**) Problem definition and (**right**) the XFEM meshes.

In this example, HF testing of a concrete material was carried out to verify the capability of the FVM-XFEM model. The geometry and the relative boundary conditions of the standard cubical concrete specimen are depicted in Figure 11 (left). The prefabricated cracks were generated by inserting a steel sheet into the mold at the center of the specimen before pouring the concrete. The sizes of the specimen and the pre-existing crack were 150 mm and 50 mm, respectively. During the experiment, the fluid pressure was directly imposed on the crack faces along the pre-existing fracture interface, and the fluid pressure was gradually increased until the specimen failed. For measuring the crack opening displacement and the fluid pressure distribution inside the fracture, strain gauges were placed from the crack tip to the top and bottom surface.

Figure 11 (right) displays the two-dimensional finite element mesh with a refined mesh near the crack-tip, which contained 6650 elements and 4587 nodes. The mesh size around the fracture was fine enough to properly capture the distribution of the fluid pressure and traction. According to the situation at the test site, the boundary conditions of this problem were set as follows: the bottom boundary of the model was fixed, whereas the other boundaries were unrestrained. In addition, a large variety of water pressure conditions, imposed on the center of the fracture, were simulated to determine the critical fluid pressure required to cause damage. The material parameters used in this numerical example were obtained by material property tests, and they are listed in Table 2.

**Figure 12:**
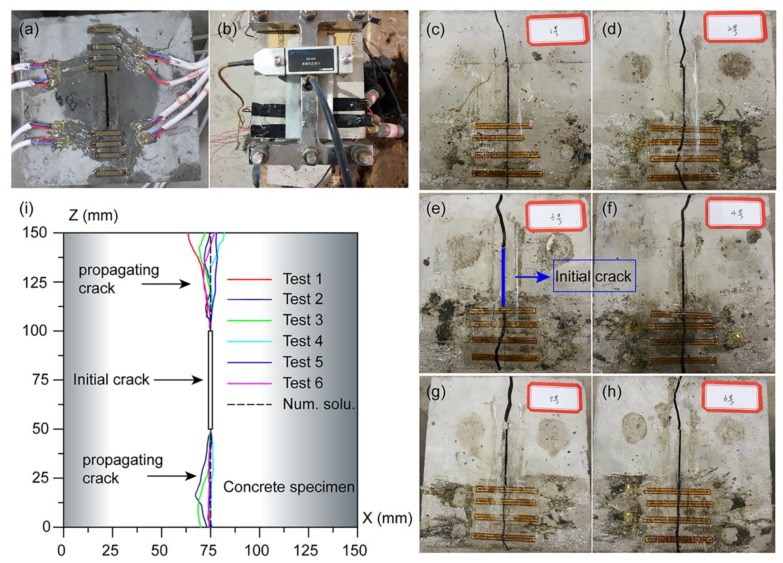
The test process (see **a**,**b**) and chart of the fracture propagation path containing both the test results (see **c**–**h**) and the numerical results (**i**).

**Figure 13:**
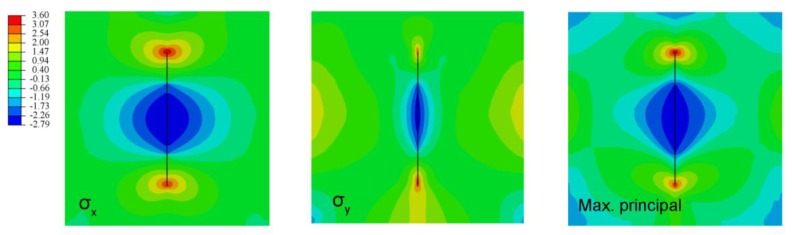
The contours of the stress distributions using FVM-XFEM (unit: MPa).

For the specimen test, when the fluid pressure across the fracture was raised to about 1.75 MPa, hydraulic fracturing failure of the specimen occurred. On the other hand, the critical fracturing pressure value predicted by the numerical solution was 1.70 MPa. The relative error between the two solutions was only 2.9%, which implies that the proposed model was both reasonable and precise. The fracture opening profiles of the tests and the numerical simulation are presented in Figure 12. For the numerical simulation, the fracture propagated along the axis until the specimen was damaged completely, whereas in the test, the direction of the propagating fracture slightly deviated from the axial direction. The reason was that the concrete material in the present model was assumed to be homogeneous and isotropic. In contrast, the test specimens were not fully isotropic, and they contained guide holes and ducts. Nevertheless, the numerical results correlated well with the testing results. The contours of the stress distribution is shown in Figure 13.

**Figure 14:**
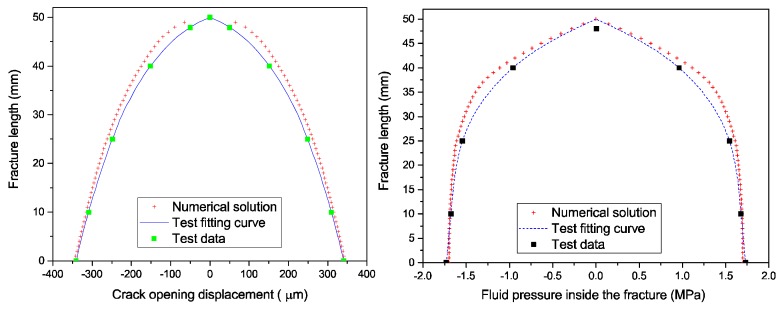
Comparison of the numerical and test results regarding: (**left**) the fracture width and (**right**) the fluid pressure distribution.

Figure 14 (left) shows the fracture growth length versus crack opening displacement curve. A remarkable difference between the present model and the test result was observed at the beginning of loading. The explanation for this is that the hydraulic loading rate of the test was limited, which resulted in a low crack propagation rate. The pressure flow, therefore, did not immediately enter the fracture. However, once the crack opening displacement approached a certain value, the fluid began to naturally flow into the fracture. Hence, as the results illustrate, the matching was very good when the fracture aperture was enlarged.

Figure 14 (right) compares the distribution of the fluid pressure across the fracture. It is visible that the fluid pressure possessed a relatively flat gradient along the fracture until it suddenly dropped to zero near the fracture tip. There was a slight discrepancy between the numerical model and the test results. The reason for this was that the concrete material was permeable, which allowed leak-off in the experiment; this resulted in the fracturing fluid dissipating into the surrounding medium. Additionally, the test results were not in good agreement with the numerical solution around the fracture tip; this could be contributed to the limitation of test conditions. The fluid pressure measuring devices were not arranged near the edges of the specimens due to limited space, which caused the non-conformity. However, in general, the analytical fluid pressure distribution calculations were basically consistent with the results predicted by the numerical model. These findings indicated the ability of the proposed model to simulate the HF of concrete materials.

### 3.3. Hydraulic Fracturing in a Concrete Gravity Dam

**Figure 15:**
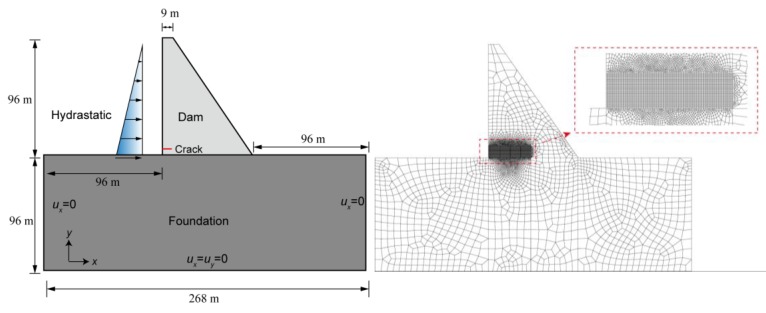
A concrete gravity dam under hydrostatic pressure: the geometry, boundary conditions (**left**), and XFEM meshes (**right**).

In the last example, the FVM-XFEM model was used to analyse the hydraulic fracturing process in a typical gravity dam, which helps illustrate the performance of the model in a more practical application. This example was initially modelled by Ren et al. [54] on the basis of the linear elastic fracture mechanics. The geometry of the dam and the corresponding boundary conditions are illustrated in Figure 15 (left). As shown in the figure, the height of the dam was 96 m, and the width of the bottom and top of the dam were 76 m and 9 m, respectively. However, it is more reasonable to also consider the foundation of the concrete dam. The foundation extended the horizontal distance of the dam by twice the height of the dam, including from the dam toe to the downstream and from the dam heel to the upstream; the foundation doubled the vertical dimension of the dam as well. The upstream face was perpendicular to the x-direction, and the slope of the downstream face had a horizontal-to-vertical ratio of 1:7. It was assumed that, during building operations, an 8 m-long and 0.02 mm-wide initial horizontal crack was generated on the upstream face of the dam, which is located 8 m above the dam heel.

A two-dimensional FEM model for simulating the HF of the dam was established, shown in Figure 15 (right). There were 6652 elements and 7812 nodes, and the size of mesh was 0.3 m × 0.3 m in the region where the fracture was expected to propagate. As far as boundary conditions are concerned, the left and right sides of the dam foundation were constrained in the *x*-direction, the bottom displacement of the foundation was set to zero in both directions, and the other boundary conditions were unrestrained. The applied loads consisted of the weight of the dam and the hydrostatic pressure in the reservoir. The water level increased from zero to the critical upstream water level 96 m with each step of 2.5 m. During this process, the initial crack will propagate. The material parameters used for the numerical simulation in this example are summarized in Table 3. The crack automatically propagated in the dam when the maximum tensile stress reached the ultimate tensile strength.

**Figure 16:**
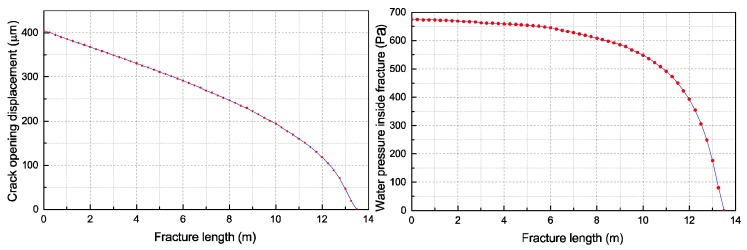
The crack mouth opening displacement (**left**) and the water pressure distribution along the crack (**right**).

Figure 16 presents the crack opening displacement along the fracture and the water pressure distribution within the crack, respectively. The fracture length corresponding to an intermediate analysis step at the upstream water level of 75 m was determined to be approximately 12 m. Clearly, the crack opening displacement gradually decreased along the fracture. Moreover, the water pressure within the crack changed mildly in the vicinity of the initial crack, whereas it varied significantly near the crack-tip zone. The numerical results were similar to the test results in the second example.

**Figure 17:**
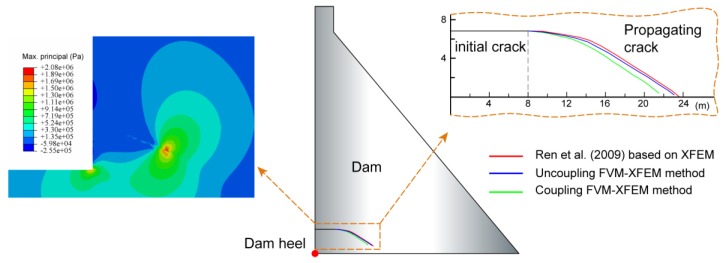
The contour of maximum principal stress using the coupling FVM-XFEM method (**left**) and the patterns of crack growth in the concrete dam using the FVM-XFEM method and the “constant pressure algorithm” (**right**).

Three crack growth paths are plotted in Figure 17, including the solutions generated by both the coupled and the uncoupled FVM-XFEM models as well as the solution by Ren et al. using the constant pressure algorithm based on the XFEM. It was worth mentioning that the coupled model allowed the water pressure inside the fracture to vary with time, while in both the uncoupled model and the constant pressure algorithm, the water pressure acting at the fracture interface was assumed to be a constant value that corresponded to the level of water in the reservoir. Obviously, these crack growth paths propagated towards the downstream and towards the bottom of the dam. In addition, one could observe that the uncoupled results predicted by the present model closely matched the numerical outcomes performed by Ren et al. using the constant pressure algorithm. The excellent correlation between the two results illustrated the capability of the proposed model in simulating the HF process in a concrete gravity dam. However, for a more realistic representation, the variation of water pressure along the fracture was considered in the coupled model. The numerical results showed that compared to the uncoupled model, the crack growth length in the coupled model was short and the extension angle was big, which confirmed the general rule.

## 4. Conclusions

In this study, a hybrid approach called FVM-XFEM was proposed for hydraulic fractures in quasi-brittle materials. The fully coupled formulation was established based on the linear momentum balance equation and the one-dimensional laminar flow model, which accounted for the coupling effect between the fluid pressure and the fracture opening width. The discontinuous fracture was captured by means of the XFEM, and the fracture process was modelled using the cohesive zone model. The different approximation spaces were obtained using the extended finite element method for the solid deformation and the finite volume method for the fluid pressure. The coupled, discretized model was solved by exploiting the Picard iteration technique.

In the first example, the KGD fracture problem in a rock formation was analyzed. The results illustrated that the numerical results were in good agreement with both the analytical solution as well as the FEM with zero-thickness elements. In the second example, a hydraulic fracturing test of the concrete material was carried out to verify our FVM-XFEM model, and the numerical outcomes were basically consistent with the test results. The differences between the results were attributed to the fact that the test conditions were limited and to the occurrence of fluid leak-off in the concrete around the crack. The validity of the FVM-XFEM model was confirmed in the first two examples; therefore, the proposed model was implemented to analyze a practical engineering problem in the last example. The HF process of a concrete gravity dam was modelled under different water pressure distributions. In this case, it was observed that the fracture can propagate in arbitrary directions. The results of the uncoupled FVM-XFEM model were quite similar to those of the constant pressure algorithm based on the XFEM, which resulted from both methods employing a constant pressure value at the fracture interface. However, a more realistic distribution of water pressure across the fracture was considered in the coupled mode. As predicted by the general rule, the numerical results produced by the coupled FVM-XFEM model exhibited a shorter crack growth length and a larger extension angle than the uncoupled FVM-XFEM model.

In summary, the hybrid FVM-XFEM model can effectively simulate hydraulic fracturing in quasi-brittle materials, such as shales for rock materials and dams composed of concrete materials.

## Figures and Tables

**Table 1:** Parameters for the bulk material and the cohesive zone (from [22]).

Young’s modulus	*E* = 17.0 GPa
Poisson’s ratio	*ν* = 0.2
Tensile strength	*f_t_* = 1.25 MPa
Cohesive fracture energy	*G_c_* = 120 N/m
Water density	*ρ_w_* = 1000 kg/m^3^
Water dynamic viscosity	*μ* = 1.00 × 10^−4^ Pa s
Fluid injection rate	*Q*_0_ = 5.00 × 10^−4^ m^2^/s

**Table 2:** Parameters of the concrete specimens used in the hydraulic fracture test.

Young’s modulus	*E* = 27.25 GPa
Poisson’s ratio	*ν* = 0.173
Tensile strength	*f_t_* = 2.01 MPa
Cohesive fracture energy	*G_c_* = 140 N/m
Water density	*ρ_w_* = 1000 kg/m^3^
Water dynamic viscosity	*μ* = 1.00 × 10^−6^ kPa s

**Table 3:** Parameter of the concrete gravity dam.

Young’s modulus	*E* = 25.0 GPa
Poisson’s ratio	*ν* = 0.167
Tensile strength	*f_t_* = 2.05 MPa
Cohesive fracture energy	*G_c_* = 150 N/m
Water density	*ρ_w_* = 1000 kg/m^3^
Water dynamic viscosity	*μ* = 1.00 × 10^−6^ kPa∙s
Solid density	*ρ_w_* = 2400 kg/m^3^

## References

[1] Detournay E. (2016). Mechanics of Hydraulic Fractures. Annu. Rev. Fluid Mech..

[2] Levasseur S., Charlier R., Frieg B., Collin F. (2010). Hydro-mechanical modelling of the excavation damaged zone around an underground excavation at Mont Terri Rock Laboratory. Int. J. Rock Mech. Min..

[3] Rahm D. (2011). Regulating hydraulic fracturing in shale gas plays: The case of Texas. Energy Policy.

[4] Adachi J.I., Detournay E. (2008). Plane strain propagation of a hydraulic fracture in a permeable rock. Eng. Fract. Mech..

[5] Perkins T.K., Kern L.R. (1961). Widths of hydraulic fractures. J. Pet. Technol..

[6] Nordgren R. (1972). Propagation of a vertical hydraulic fracture. SPE J..

[7] Geertsma J., de Klerk F. (1969). A rapid method of predicting width and extent of hydraulically induced fractures. J. Pet. Technol..

[8] Khristianovic S., Zheltov Y. Formation of vertical fractures by means of highly viscous liquid. Proceedings of the Fourth World Petroleum Congress.

[9] Bazant Z.P. Pore Pressure, uplift, and failure analysis of concrete dams. Proceedings of the Numerical Analysis of Dams International Symposium.

[10] Bazant Z.P. (1979). Stability and post-critical growth of a system of cooling or shrinkage cracks. Int. J. Fract..

[11] Bazant Z.P., Ohtsubo H. (1977). Stability conditions for propagation of a system of cracks in a brittle solid. Mech. Res. Commun..

[12] Brühwiler E., Saouma V.E. (1995). Water fracture interaction in concrete—Part II: Hydrostatic pressure in cracks. ACI Mater. J..

[13] Gan L., Zhang K.L., Zhang H.W., Tian Z.Y. (2017). Coupling analysis of hydraulic fracturing computation base on element-free method. Cluster Comput..

[14] Miehe C., Hofacker M., Welschinger F. (2010). A phase field model for rate-independent crack propagation: Robust algorithmic implementation based on operator splits. Comput. Meth. Appl. Mech. Eng..

[15] Paggi M., Corrado M., Reinoso J. (2018). Fracture of solar-grade anisotropic polycrystalline Silicon: A combined phase field–cohesive zone model approach. Comput. Meth. Appl. Mech. Eng..

[16] Nguyen T.H.A., Bui T.Q., Hirose S. (2018). Smoothing gradient damage model with evolving anisotropic nonlocal interactions tailored to low-order finite elements. Comput. Meth. Appl. Mech. Eng..

[17] Boone T., Ingraffea A. (1990). A numerical procedure for simulation of hydraulically-driven fracture propagation in poroelastic media. Int. J. Numer. Anal. Methods Geomech..

[18] Schrefler B.A., Secchi S., Simoni L. (2006). On adaptive refinement techniques in multi-field problems including cohesive fracture. Comput. Meth. Appl. Mech. Eng..

[19] Secchi S., Schrefler B.A. (2012). A method for 3-D hydraulic fracturing simulation. Int. J. Fract..

[20] Sarris E., Papanastasiou P. (2011). The influence of the cohesive process zone in hydraulic fracturing modelling. Int. J. Fract..

[21] Segura J.M., Carol I. (2004). On zero-thickness interface elements for diffusion problems. Int. J. Numer. Anal. Methods Geomech..

[22] Carrier B., Granet S. (2012). Numerical modeling of hydraulic fracture problem in permeable medium using cohesive zone model. Eng. Fract. Mech..

[23] Belytschko T., Krongauz Y., Organ D., Fleming M., Krysl P. (1996). Meshless methods: An overview and recent developments. Comput. Meth. Appl. Mech. Eng..

[24] Lucy L.B. (1977). A numerical approach to the testing of the fission hypothesis. Astron J..

[25] Cundall P.A., Strack O.D.L. (1979). A discrete numerical model for granular assemblies. Géotechnique.

[26] Melenk J.M., Babuska I. (1996). The partition of unity finite element method: Basic theory and applications. Comput. Meth. Appl. Mech. Eng..

[27] Aliabadi M.H., Brebbia C.A. (1993). Advanced Formulations in Boundary Element Methods.

[28] Ganis B., Mear M.E., Sakhaee-Pour A., Wheeler M.F., Wick T. (2014). Modeling fluid injection in fractures with a reservoir simulator coupled to a boundary element method. Comput. Geosci..

[29] Belytschko T., Black T. (1999). Elastic crack growth in finite elements with minimal remeshing. Int. J. Numer. Methods Eng..

[30] Moës N., Dolbow J., Belytschko T. (1999). A finite element method for crack growth without remeshing. Int. J. Numer. Methods Eng..

[31] Moës N., Belytschko T. (2002). Extended finite element method for cohesive crack growth. Eng. Fract. Mech..

[32] Zi G., Belytschko T. (2003). New crack-tip elements for XFEM and applications to cohesive cracks. Int. J. Numer. Methods Eng..

[33] Liu F.S., Gordon P., Meier H., Valiveti D. (2017). A stabilized extended finite element framework for hydraulic fracturing simulations. Int. J. Numer. Anal. Methods Geomech..

[34] Jiang S.Y., Du C.B. (2017). Coupled Finite Volume Methods and Extended Finite Element Methods for the Dynamic Crack Propagation Modelling with the Pressurized Crack Surfaces. Shock Vib..

[35] Song J.H., Areias P.M.A., Belytschko T. (2006). A method for dynamic crack and shear band propagation with phantom nodes. Int. J. Numer. Methods Eng..

[36] Zamani A., Eslami M.R. (2010). Implementation of the extended finite element method for dynamic thermoelastic fracture initiation. Int. J. Solids Struct..

[37] De Borst R., Réthoré J., Abellan M.A. (2006). A numerical approach for arbitrary cracks in a fluid-saturated medium. Arch. Appl. Mech..

[38] Réthoré J., de Borst R., Abellan M.A. (2007). A two-scale approach for fluid flow in fractured porous media. Int. J. Numer. Methods Eng..

[39] Mohammadnejad T., Khoei A.R. (2013). Hydro-mechanical modeling of cohesive crack propagation in multiphase porous media using the extended finite element method. Int. J. Numer. Anal. Methods Geomech..

[40] Shi L., Yu T., Bui T.Q. (2015). Numerical Modelling of Hydraulic Fracturing in Rock Mass by Xfem. Soil Mech. Found. Eng..

[41] Zhang X., Bui T.Q. (2015). A fictitious crack XFEM with two new solution algorithms for cohesive crack growth modeling in concrete structures. Eng. Comput..

[42] Wang K.F., Zhang Q., Xia X.Z., Wang L., Liu X.C. (2015). Analysis of hydraulic fracturing in concrete dam considering fluid-structure interaction using XFEM-FVM model. Eng. Fail. Anal..

[43] Zhou L., Gou Y., Hou Z.M., Were P. (2015). Numerical modeling and investigation of fracture propagation with arbitrary orientation through fluid injection in tight gas reservoirs with combined XFEM and FVM. Environ. Earth Sci..

[44] Batchelor G.K. (1967). An Introduction to Fluid Dynamics.

[45] Versteeg H.K., Malalasekera W. (1995). An Introduction to Computational Fluid Dynamics: The Finite Volume Method.

[46] Witherspoon P.A., Wang J.S.Y., Iwai K., Gale J.E. (1980). Validity of cubic law for fluid flow in a deformable rock fracture. Water Resour. Res..

[47] Wells G.N., Sluys L.J. (2001). A new method for modelling cohesive cracks using finite elements. Int. J. Numer. Methods Eng..

[48] Hillerborg A., Mod’eer M., Petersson P.E. (1976). Analysis of crack formation and crack growth in concrete by means of fracture mechanics and finite elements. Cem. Concr. Res..

[49] Dias-da-Costa D., Alfaiate J., Sluys L.J., Julio E. (2010). A comparative study on the modelling of discontinuous fracture by means of enriched nodal and element techniques and interface elements. Int. J. Fract..

[50] Liu W., Yang Q.D., Mohammadizadeh S., Su X.Y. (2014). An efficient augmented finite element method for arbitrary cracking and crack interaction in solids. Int. J. Numer. Methods Eng..

[51] Fries T.P., Baydoun M. (2012). Crack propagation with the extended finite element method and a hybrid explicit-implicit crack description. Int. J. Numer. Methods Eng..

[52] Rabczuk T., Zi G., Gerstenberger A., Wall W.A. (2008). A new crack tip element for the phantom-node method with arbitrary cohesive cracks. Int. J. Numer. Methods Eng..

[53] Khoei A.R., Vahab M., Haghighat E., Moallemi S. (2014). A mesh-independent finite element formulation for modeling crack growth in saturated porous media based on an enriched-FEM technique. Int. J. Fract..

[54] Ren Q.W., Dong Y.W., Yu T.T. (2009). Numerical modeling of concrete hydraulic fracturing with extended finite element method. Sci. China-Technol. Sci..

